# Practical Notes on Diagnosis and Treatment

**Published:** 1909-06-26

**Authors:** 


### Practical Notes on Diagnosis and Treatment.

### The Schafer Method of Artificial Respiration.

The operator kneels by the side of or across the prone patient, places his hands over the lower ribs, and swings his body forwards and backwards so as to allow his weight alternately to fall vertically on the wrists and to be removed. The pressure is exerted gradually and slowly for three seconds, then relaxed for two seconds: and so on twelve times a minute.

### Attempted Suicide.

In the treatment of attempted suicide special care and watchfulness must be exercised to prevent a further and more successful attempt. Such an attempt is a common-law misdemeanour only, and hence there is no need to report the facts to the police; a constable cannot arrest the victim without a warrant unless the affair took place under his eyes.—*Dr. S. B. Atkinson.*

### Bullet Wounds in War.

The great majority of small-bore bullet-wounds may be considered aseptic in the first instance, except where the bullet has carried in portions of clothing with it, or has opened up a septic region, as the intestine. Infection takes place in too many cases from interference by the surgeon or others. Plugging wounds for hæmorrhage is a sure way of infecting them.—*Major Spencer.*

### Hysteria.

I believe it would not be untrue to say that if anybody wished to invent a new sign or "stigma" for hysteria, he could do it by merely suggesting the same symptom in every patient. If, for example, he said in the presence of his hysterical patients that such cases have always anæsthesia to temperature of the big toe of the left foot, he would certainly find such anæsthesia present.—*Dr. A. F. Hertz.*

